# Expression, Purification and Refolding of a Human Na_V_1.7 Voltage Sensing Domain with Native-like Toxin Binding Properties

**DOI:** 10.3390/toxins13100722

**Published:** 2021-10-12

**Authors:** Ryan V. Schroder, Leah S. Cohen, Ping Wang, Joekeem D. Arizala, Sébastien F. Poget

**Affiliations:** 1 Department of Chemistry, College of Staten Island, University of New York, 2800 Victory Blvd., Staten Island, NY 10314, USA; rschroder92@gmail.com (R.V.S.); Leah.Cohen@csi.cuny.edu (L.S.C.); ping.wang@cix.csi.cuny.edu (P.W.); jarizala@gradcenter.cuny.edu (J.D.A.); 2 The Ph.D. Program in Biochemistry, The Graduate Center of the City University of New York, 365 Fifth Avenue, New York, NY 10016, USA; 3 The Ph.D. Program in Chemistry, The Graduate Center of the City University of New York, 365 Fifth Avenue, New York, NY 10016, USA

**Keywords:** membrane protein refolding, voltage-gated sodium channel, voltage sensor, bacterial expression of mammalian proteins, peptide toxin, lipid reconstitution

## Abstract

The voltage-gated sodium channel Na_V_1.7 is an important target for drug development due to its role in pain perception. Recombinant expression of full-length channels and their use for biophysical characterization of interactions with potential drug candidates is challenging due to the protein size and complexity. To overcome this issue, we developed a protocol for the recombinant expression in *E. coli* and refolding into lipids of the isolated voltage sensing domain (VSD) of repeat II of Na_V_1.7, obtaining yields of about 2 mg of refolded VSD from 1 L bacterial cell culture. This VSD is known to be involved in the binding of a number of gating-modifier toxins, including the tarantula toxins ProTx-II and GpTx-I. Binding studies using microscale thermophoresis showed that recombinant refolded VSD binds both of these toxins with dissociation constants in the high nM range, and their relative binding affinities reflect the relative IC_50_ values of these toxins for full-channel inhibition. Additionally, we expressed mutant VSDs incorporating single amino acid substitutions that had previously been shown to affect the activity of ProTx-II on full channel. We found decreases in GpTx-I binding affinity for these mutants, consistent with a similar binding mechanism for GpTx-I as compared to that of ProTx-II. Therefore, this recombinant VSD captures many of the native interactions between Na_V_1.7 and tarantula gating-modifier toxins and represents a valuable tool for elucidating details of toxin binding and specificity that could help in the design of non-addictive pain medication acting through Na_V_1.7 inhibition.

## 1. Introduction

Voltage-gated sodium channels (VGSCs) are ion channels that are essential for the initiation and propagation of action potentials in excitatory cells [1]. Their central ion-conducting α subunit is made of a single polypeptide chain with two functional parts: the voltage sensing domains (VSDs) and the pore domain that forms the sodium-selective pore. The VSDs are comprised of the S1–S4 transmembrane helices of each of the pseudotetrameric channel’s four repeating units [2]. Na_V_1.7 is a VGSC isoform of therapeutic interest since it has been shown to be involved with pain response [3]. Molecules that bind to and inhibit this channel could potentially be used as pain therapeutics without addictive side effects.

Many animals have developed toxins that target VGSCs. Such toxins can offer great insight into channel function and selective modulation due to their large number and different binding specificities and modes of function [1]. In particular, several gating-modifier toxins have been identified that bind to the VSDs of Na_V_1.7 with high affinity and specificity [4]. Among them, the tarantula-derived ProTx-II is one of the most widely studied toxins that binds to and inhibits the function of Na_V_1.7. ProTx-II is a peptide toxin that adopts an inhibitor cystine knot (ICK) motif. Through interaction with the VSDs, it prevents the channel from adopting the open, ion-conducting conformation [5]. The recent complex structures of ProTx-II with a Na_V_1.7-bacterial sodium channel chimera obtained by X-ray crystallography [6] and with full-length human Na_V_1.7 obtained by cryo-electron microscopy (cryo-EM) [7] represent important steps toward elucidating the toxin–channel interactions with the goal of exploiting these toxins as lead compounds for rational drug design. However, in the X-ray crystal structure, only the extracellular facing segments of the chimera VSD correspond to the human sequence, and the EM structures are of low resolution in the VSD–toxin interaction region. Therefore, the need persists for additional studies using different experimental approaches and more native-like constructs to refine our understanding of the toxin–VSD interactions.

GpTx-I is another tarantula ICK toxin that has been shown to selectively bind to Na_V_1.7 [8]. GpTx-I is of particular interest because it exhibits analgesic effects in mice [9]. Therefore, it would be beneficial to obtain structural information on GpTx-I–Na_V_1.7 interactions to uncover determinants of relative specificity and binding affinity for the channel.

For the cryo-EM study of full-length Na_V_1.7 in complex with ProTx-II, large-scale transfected mammalian cell growths (16 L) were needed to obtain sufficient protein for structure determination [7]. Therefore, it would be beneficial to develop a higher-yield bacterial expression and purification procedure to obtain functional channel domains for characterizing toxin–channel interactions. Using other biophysical techniques in addition to cryo-EM and X-ray crystallography could also reveal complementary structural information on toxin binding but might require labeling strategies that are difficult to achieve in mammalian cell culture. In this study, we designed and validated an *E. coli* expression and refolding procedure that yields the human Na_V_1.7 VSD of repeat II (VSD2) in a native-like conformation capable of replicating toxin binding properties displayed by the full-length wild-type channel. Since VSD2 is the known interaction site for many inhibiting gating-modifier toxins, we expect that this construct will be useful in the identification of new drug lead compounds and in the structural and biophysical characterization of the binding interactions between already known and newly discovered toxins and the Na_V_1.7 VSD2.

Inclusion body expression followed by refolding of a membrane protein domain was previously utilized by Devaraneni and co-workers to produce functionally active KvAP, a bacterial voltage-gated potassium channel [10], but this method has not yet been applied to the production of a human voltage-gated ion channel or VSD. Additionally, the VSD of repeat II of Na_V_1.4 has been produced through cell-free methods and was subsequently used for biophysical characterization by solution-state NMR [11]. However, cell-free expression is costly to scale up, especially when isotope-labeled amino acids need to be incorporated, which can be much more easily and economically achieved by the method we present here.

## 2. Results

### 2.1. Expression and Purification of Na_V_1.7 VSD2

**Figure 1:**
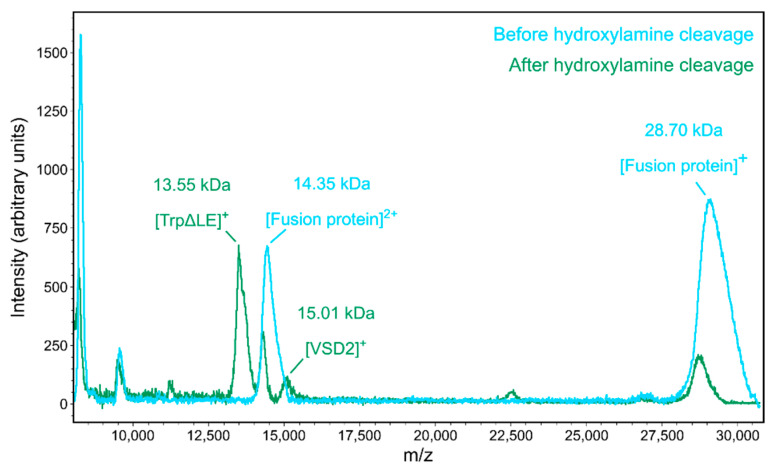
MALDI mass spectra of the fusion protein before and after incubation with hydroxylamine. Before cleavage, only the intact fusion protein (theoretical mass 28,699 Da) is observed. After overnight incubation with 1.1 M hydroxylamine, the intensity of fusion protein is significantly reduced and peaks for the cleaved voltage sensing domain II (VSD2, theoretical mass 14,982 Da) and TrpΔLE fragment (theoretical mass 13,621 Da) become visible.

Direct expression of the isolated VSD2 into the bacterial membrane showed very low recombinant protein yields, so we instead performed insoluble expression followed by refolding of the VSD. The Na_V_1.7 VSD2 was cloned into a pSW02 vector, which contains a TrpΔLE fusion partner that drives the recombinant protein into inclusion bodies [12,13]. Because the inclusion body resolubilization requires harsh denaturing conditions, proteolytic cleavage of the VSD was not feasible. We instead used hydroxylamine, which cleaves peptides between asparagine and glycine residues in a variety of conditions, including in the presence of denaturing agents [14,15]. The cleavage of the fusion protein was successful in a solution of 1% w/v *N*-lauryl sarcosine, 6 M guanidine HCl and 1.1 M hydroxylamine, as confirmed by MALDI mass spectrometry (Figure 1). A small portion of uncleaved fusion protein remained after ~5 days of cleavage and was removed by size exclusion chromatography (Supplementary Material, Figure S1). While the MALDI mass spectrometry peak corresponding to the VSD had lower intensity compared to the other species, this must be due to its lower ionization efficiency, since Coomassie-stained SDS-PAGE gel analysis after hydroxylamine cleavage showed similar concentrations of cleaved VSD and TrpΔLE fragments and only a small fraction of uncleaved fusion protein was present (Figure S1).

Following purification in the denatured state, the VSD was reconstituted into phospholipids. Successful reconstitution was achieved in a two-step procedure where the denatured VSD was first exchanged into sodium dodecyl sulfate (SDS) micelles followed by reconstitution into dimyristoyl phosphatidylcholine (DMPC) lipids. DMPC was added to the SDS solution with a 1:300 protein/lipid ratio, and the SDS was removed by dialysis against water for 1–2 weeks with several solvent changes. A yield of ~2 mg of VSD per liter of cell growth was obtained using this method.

**Figure 2:**
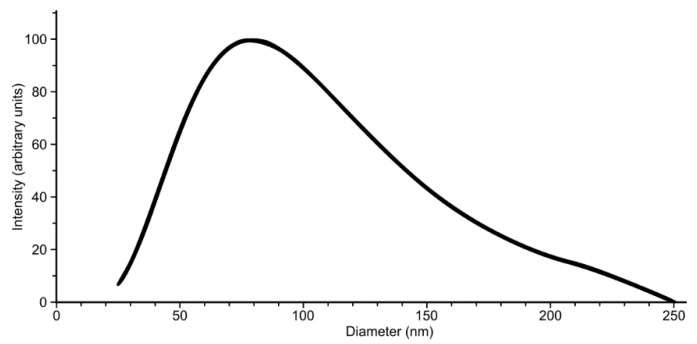
Dynamic light scattering (DLS) analysis of Na_V_1.7 VSD2 after dimyristoyl phosphatidylcholine (DMPC) reconstitution shows a size distribution with a maximum around 80 nm diameter particles. This is consistent with the expected size of small unilamellar vesicles (SUVs).

Even after extensive dialysis, the reconstitution solution was optically clear, indicating reconstitution into small unilamellar vesicles (SUVs). Because no remaining SDS was detectable in solution by proton NMR and considering that the VSD peptide on its own precipitates in aqueous solution, we concluded that the protein was inserted into these SUVs. To further characterize the nature of the VSD2/DMPC complex, we used dynamic light scattering (DLS). The DLS data show that the complex has a mean diameter of roughly 80 nm (Figure 2), which is consistent with the presence of SUVs. Previous experiments have shown that unilamellar vesicles of 30–100 nm diameter can be obtained by detergent removal from lipid–detergent mixtures [16]. Interestingly, the formation of SUVs was dependent on the slow exchange achieved by using a low-molecular-weight cutoff (3.5 kDa) dialysis membrane in the SDS removal step. When a 30 kDa cutoff membrane was used, we obtained turbid solutions after dialysis, indicating the formation of much larger particles.

We next performed circular dichroism (CD) spectroscopy on the VSD2/DMPC complex to determine the secondary structure of the refolded channel domain. The CD spectrum shows the characteristic minima at 208 and 222 nm, indicating the presence of α-helical secondary structure as expected for VSD2 based on the cryo-EM structure of full-length Na_V_1.7 [7] (Supplementary Material, Figure S2).

### 2.2. Toxin Binding Studies

**Figure 3:**
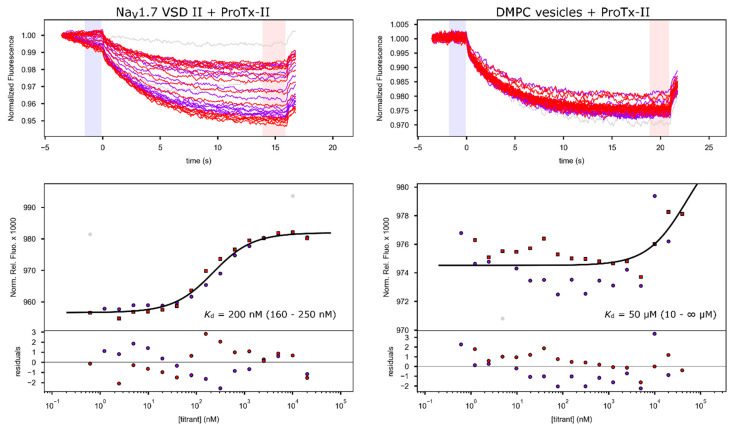
Microscale thermophoresis (MST) binding curves for fluorescently labeled ProTx-II titrated with Na_V_1.7 VSD2 in DMPC (left) and with empty DMPC vesicle control (right). The top panels show raw fluorescent time traces of two independent titrations (in red and blue, respectively), and the bottom panels show the normalized fluorescence data with the best fit as well as the resulting residuals. The resulting dissociation constants with the 63.8% confidence interval limits in brackets are shown. The light blue and red shaded areas in the raw fluorescence graph indicate the regions used to average pre- and post-temperature jump fluorescence data used to calculate the relative fluorescence intensities plotted in the lower graphs. DMPC vesicles for the control experiments were prepared following the same method as for voltage sensing domain (VSD) reconstitution (starting from SDS-solubilized lipids), except without the addition of protein, and the concentrations used in the fit are equivalent to what the protein concentration would have been at the same protein–lipid ratio as used in the VSD refolding. Therefore, the apparent *K*_d_ resulting from the negative control can be directly compared to that of the VSD experiment.

As an indirect confirmation that the refolded protein adopts a native-like tertiary structure, we assayed the ability of the recombinant VSD2 to bind tarantula peptide toxins known to affect the full-length channel. We used microscale thermophoresis (MST) [17] to determine the binding affinity between the reconstituted VSD2/DMPC complex and the tarantula toxins ProTx-II and GpTx-I. In both cases, the toxins were labeled with Alexa Fluor^TM^ 488 (Invitrogen, Carlsbad, CA, USA) via NHS–ester coupling to amino groups on the toxin. Mass spectrometry analysis of the labeling reactions showed that the predominant labeled species were derivatized with a single fluorescent dye molecule (Supplementary Material, Figure S3). Considering that the amino terminus is the more reactive amine due to its lower p*K*_a_, we assume that the labeled toxin molecules retain mostly underivatized side-chain amines, which is important considering that some of these amines have previously been implicated in channel binding [1]. ProTx-II was recombinantly produced [18], and binding experiments were conducted by titrating VSD2/DMPC into 50 nM fluorescently labeled ProTx-II (Figure 3). The experiment was repeated twice and yielded a *K*_d_ value of 200 nM after fitting of the combined binding data (63.8% confidence interval: 160–250 nM). All results and parameters for the binding fits are summarized in Table 1. 

A control experiment consisting of a titration with empty DMPC vesicles with an equivalent amount of lipids present compared to the VSD titration resulted in much weaker binding with an apparent *K*_d_ of 50 µM (10 µM–∞, Figure 3), indicating that the observed binding results from specific VSD–toxin interactions and not simply from the association of the toxin with the lipid membrane.

**Figure 4:**
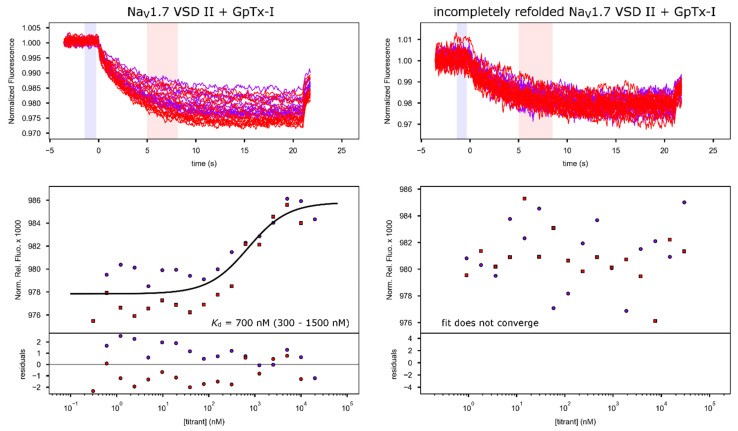
MST binding curves for GpTx-I binding to fully folded as well as incompletely refolded Na_V_1.7 VSD2 in DMPC vesicles. In each case, VSD in DMPC solution was titrated into 50 nM of fluorescently labeled GpTx-I. Data are presented as in Figure 3.

Reported IC_50_ values for ProTx-II inhibition of Na_V_1.7 are in the range of 0.3 to 3 nM [5,6,20]. Even though *K*_d_ values cannot be directly compared to channel inhibition IC_50_ values [21], the fact that the *K*_d_ values we measured are two to almost three orders of magnitude higher than that the reported IC_50_ values nevertheless indicates that toxin binding affinity of our refolded, isolated VSD was lower than for full channels in native membranes. Such a reduction in affinity is not surprising for a number of reasons: First, small DMPC vesicles do not perfectly mimic the native membrane as they do not have the same lipid distribution and surface curvature. These factors are important because tarantula gating-modifier toxin interaction with the membrane lipids is known to contribute to overall activity [22,23]. Second, while the interaction of ProTx-II with VSD2 is the most important contributor to toxin activity, interactions with other parts of the channel could also contribute to the overall mechanism of action (for example, in the cryo-EM structure of Na_V_1.7 with ProTx-II, electron density for a bound toxin is also found adjacent to VSD4 [7]). In addition, the presence of residual unlabeled toxin, which may have higher binding affinity and thus could out-compete the fluorescently labeled peptide, could also lead to a higher measured *K*_d_, as could the presence of a small fraction of toxins labeled on side-chain amines involved in binding. As an additional control to rule out that the observed binding is merely due to the interaction of the toxin with the lipids and random membrane-embedded amino acids that would be present in the proteoliposome even if the VSD were not properly refolded, we also produced a control sample where we reduced the dialysis time for DMPC refolding fivefold. While this still yielded lipid-embedded protein as shown by an absorption peak at 280 nm present in the lipid preparation (Supplementary Material, Figure S4), this protein sample exhibited toxin binding with an affinity too low for any reliable fit (binding experiment done with GpTx-I, Figure 4), indicating that the shortened refolding time was not sufficient for the protein to adopt its native conformation and thus display high-affinity toxin binding. This demonstrates that the correctly refolded protein is necessary for toxin binding and that the *K*_d_ of toxin binding measured here is indeed indicative of the fact that the refolded VSD adopts a conformation that closely resembles that of VSD2 in the full channel in a membrane.

We performed further quantitative binding studies with GpTx-I, which was prepared using FMOC peptide synthesis and oxidative refolding [8]. MST was performed with Na_V_1.7 VSD2/DMPC titrated into fluorescently labeled GpTx-I (Figure 4). Fitting of the MST binding data resulted in a *K*_d_ of 700 nM (300–1500 nM) for GpTx-I, which is 3.5 times the *K*_d_ measured for ProTx-II. Interestingly, reported IC_50_ values for Na_V_1.7 inhibition by ProTx-II and GpTx-I obtained from an identical assay were 3 and 10 nM, respectively [8]. Therefore, the ratio of the *K*_d_ values that we found for the binding of the two toxins to refolded VSD2 was essentially the same as the ratio of IC_50_ values of the two toxins against full-length Na_V_1.7 in an in vivo assay. This is compelling evidence that the refolded VSD interacts with the toxins in a way that is very similar to the interactions made in the full channel.

Additionally, an initial investigation of residues involved in GpTx-I binding was performed as a proof of concept to show that our refolded VSD can be used to elucidate specific binding characteristics of gating-modifier toxins. Mutations were chosen based on the hypothesis that GpTx-I binds similarly to ProTx-II. The mutations F813A and D816A in VSD2 were designed based on the structure of Na_V_1.7 chimera in complex with ProTx-II [6] (Supplementary Material, Figure S5). In addition, residue F813 has also been shown to be important for ProTx-II binding through site-directed mutagenesis in the whole channel [20]. After expressing and reconstituting the mutant VSDs, the same MST binding assays were performed with GpTx-I. Both mutations resulted in a substantial reduction in binding affinity, to 1.3 μM (0.6–3.0 µM) for F813A and 5.0 μM (3.0–12.0 µM) for D816A, respectively (Supplementary Material, Figure S6). This reduction in binding affinity confirms that similar residues are involved in the interaction with GpTx-I as compared to ProTx-II. Based on the relative reductions in binding affinities for the two mutants, these results suggest that the electrostatic interactions between toxin and D816 appear to have a stronger contribution to overall toxin binding than the hydrophobic interactions with F813. It also shows that our recombinant VSD is a good model system to quantitatively study the factors that directly affect binding, which will allow for more detailed comparisons between different toxin interaction sites and mechanisms to be carried out. Lastly, it is additional confirmation that our recombinant system captures details of the interactions that correspond to those with full-length channel in native membranes.

## 3. Discussion

The ability of isolated VSDs to bind gating-modifier toxins has been demonstrated in several previous studies. For example, isolated VSD from the archaebacterial potassium channel KvAP was used to pull down gating-modifier toxins from crude tarantula venom [24]. Further NMR studies were then conducted with the isolated VSD of that channel to elucidate structural details of the channel–toxin complex [25,26]. Moving from a bacterial model system to human channels, this approach has been used on isolated VSDs of the sodium channel Na_V_1.4 to elucidate structural details of the interaction between the VSD of repeat I and the spider toxin Hm-3 [27]. Recently, in a computational study to predict changes in binding affinity of different gating-modifier toxin mutants for Na_V_1.7, the authors were able to achieve binding energies in good agreement with experimental values using only the isolated VSD2 in an explicit membrane for their computer modeling [28]. All these results underscore the potential utility of the method we present here for the recombinant production of functional Na_V_1.7 VSD2 in the functional and structural analysis of channel–toxin interactions and the discovery of new toxins.

We believe that the basis of the successful refolding we achieved relies mostly on the slow exchange of the VSD from micelles into a bilayer environment. The importance of this step is clear from our refolding experiments conducted on this VSD. For instance, we also attempted to reconstitute the VSD from SDS micelles directly into various detergents such as dodecyl-phosphocholine (DPC) as well as bicelles comprised of DMPC/DHPC (dihexanoyl-phosphocholine) at various molar ratios, all of which resulted in precipitation of the VSD. However, when the VSD was exchanged into DMPC first, subsequent extraction into detergent micelles was possible, even after the removal of a majority of the DMPC. These findings indicate that the exchange of the VSD into solubilized DMPC from SDS micelles is the crucial step in the refolding of the VSD.

Lastly, this method of VSD production could also be more broadly applicable to other ion channel VSDs, opening up a large range of additional drug targets that could be addressed. The method may also enable structural studies of VSDs from various ion channel superfamilies that could shed light on their different functional roles. For example, the cyclic nucleotide-gated channels are only weakly gated by membrane voltage under most conditions even though they contain homologous VSDs [29], and structural analysis of these domains in comparison to VSDs from voltage-gated ion channels enabled by our expression method might help better understand the structural underpinning of these differences. The use of the *N*-terminal TrpΔLE fusion tag that drives the resulting fusion protein into inclusion body fractions ensures a high yield of fusion protein. One major challenge of using such expression systems to produce membrane proteins is the cleavage of the fusion tag, since enzymatic cleavage is usually not possible in the denaturing resolubilization conditions. Here, we have overcome this problem by using hydroxylamine, which has worked well for us in denaturing conditions and should be generally applicable as long as the target membrane protein does not contain a native asparagine–glycine sequence segment. With final yields of ~2 mg of refolded VSD per liter of *E. coli* in minimal medium, this method offers a promising approach to obtain VSDs for biophysical studies in a cost- and time-effective way, which may work with small adaptations (such as the type(s) of refolding lipids) for a large number of different ion channel VSDs and other small membrane proteins or protein domains.

In summary, we have designed a methodology to produce native-like folded Na_V_1.7 VSD2 with high yield. This system opens up possibilities for detailed biophysical studies of binding interactions with toxins and other drug candidates for the development of new non-addictive pain therapeutics targeting Na_V_1.7. In addition, because the protein is expressed in *E. coli*, it also enables the preparation of isotope-labeled samples or the introduction of unnatural amino acids for structural and functional studies. In addition, we used this system to begin investigating the GpTx-I binding site on Na_V_1.7 and identified two residues that are crucial for binding. Given that GpTx-I has been found to exhibit analgesic effects superior to morphine in mice and could, therefore, be an important lead compound in the development of novel pain therapeutics [9], our results underscore the utility and promise of our expression and reconstitution protocol for mechanistic studies and drug development. Lastly, the refolded VSD could also be used as a pull-down agent in the discovery of novel toxins that bind to Na_V_1.7 and could serve as a drug lead in the development of new pain therapeutics.

## 4. Materials and Methods

### 4.1. Cloning and Expression of VSD2

A gene encoding for Na_V_1.7 VSD2 was purchased from Invitrogen Life Sciences. The gene encodes for the Na_V_1.7 (UniProt ID Q15858-3) residues 732–860 and was codon-optimized for *E. coli* expression. The gene was cloned into the modified pSW02 vector [13]. The resulting fusion protein contains, from N- to C-terminus, a TrpΔLE fusion tag, a 6xHis-tag, a double hydroxylamine cleavage site (NGNG) and the VSD sequence. Expression was performed in C43(DE3) *E. coli* cells [30] in M63 minimal medium. Cells were lysed by sonication. The resulting cell lysate solution was spun down at 34,000× *g*. Pellets were re-suspended in 5 mL of 1% Triton X-100 solution followed by sonication and centrifugation at 34,000× *g* to wash the inclusion bodies. A final inclusion body wash was done in the same way but using dH_2_O. The resulting inclusion body pellets were solubilized with 50 mL per liter of cell growth of 8 M urea, 1% *N*-lauryl sarcosine by incubation at room temperature for 16 h. The fusion protein was then purified using Ni^2+^-Sepharose resin (Cytiva, Marlborough, MA, USA). After loading the fusion protein onto the column in solubilization buffer, the column was washed with 20 column volumes of 20 mM TRIS pH 8.0, 100 mM NaCl, 50 mM imidazole and 1% *N*-lauryl sarcosine, and the protein was eluted with 10 column volumes of 20 mM TRIS pH 8.0, 100 mM NaCl, 400 mM imidazole and 1% *N*-lauryl sarcosine.

### 4.2. Cleavage and Reconstitution of the VSD

The presence and purity of the fusion protein in the elution fractions of the Ni^2+^ affinity purification were confirmed by SDS PAGE using a 16% acrylamide gel. The fractions containing fusion protein were pooled and solid guanidine HCl was added to a concentration of 6 M. An equivalent volume of 2.2 M hydroxylamine HCl, 1% *N*-lauryl sarcosine and 6 M guanidine HCl at pH 8.8–9.0 was added to the pooled fractions. The resulting solution was mixed, and the cleavage reaction was allowed to proceed for a minimum of 72 h at 45 °C. The reaction mixture was then dialyzed against 1 L of 8 M urea with 1% SDS (to prevent the cleavage product from precipitating). After overnight dialysis, the cleaved proteins were dialyzed against 1 L of 1% SDS to remove the urea, and at this stage the VSD remained in solution in SDS. Size exclusion chromatography (SEC) was then performed using a Superdex 200 10/300 GL column (Cytiva, Marlborough, MA, USA) in 1% SDS with 50 mM TRIS pH 8.0 to separate the unreacted fusion protein from the cleaved VSD. The VSD-containing fractions were then pooled and used directly for refolding with DMPC. The concentration of the protein solution was measured, DMPC powder was added in a 1:300 protein/lipid molar ratio and the solution was sonicated until the lipids were dissolved. The protein/DMPC solution was then dialyzed against 2 L of dH_2_O to remove the SDS using a 3.5 kDa cutoff regenerated cellulose dialysis membrane tube. This dialysis continued for 1–2 weeks with multiple buffer exchanges. The TrpΔLE fragment precipitated at this stage and was removed by filtration with a 0.2 μm syringe filter, leaving the pure VSD/DMPC complex in solution.

### 4.3. Mass Spectrometry

MALDI-TOF mass spectrometry analysis of toxins and VSDs was performed using the ultrathin-layer sample preparation technique [31]. Samples were diluted without further purification in a 1:20 ratio into a saturated solution of α-cyano-4-hydroxycinnamic acid in 3:1:2 formic acid:water:isopropanol (for samples in detergent/lipids) or in 1:1 water:acetonitrile with 0.1% TFA (aqueous samples) and spotted onto a MALDI target pre-coated with an ultrathin layer of the same matrix. As soon as the sample showed a homogenous crystal layer, the leftover drop was removed, and the crystallized layer was washed with 5 µL of 0.1% trifluoroacetic acid (TFA) for a few seconds. MALDI-TOF spectra were recorded on a Bruker microFlex mass spectrometer (Bruker, Billerica, MA, USA) operating in linear, delayed extraction mode.

### 4.4. Circular Dichroism Spectroscopy

The CD spectra of the peptides were recorded on an AVIV model 62-DS CD instrument (AVIV Associates, Lakewood, NJ). Quartz cuvettes with path length of 0.2 mm were used. All spectra were obtained by averaging 3–5 scans in a spectral window from 260 to 185 nm at an interval of 1 nm, slit width 1 or 2 nm, with a 5 s integration time at each wavelength. CD spectra on blanks, using the DMPC solution prepared in the same way except without protein, were collected at the same instrumental conditions and subtracted from the spectra containing the VSD at a concentration of ~40 μM. CD intensities are expressed as mean residue ellipticities (deg cm^2^ dmol^−2^). Experiments were all conducted at 25 °C.

### 4.5. Dynamic Light Scattering

A Brookhaven NanoBrook series instrument was used to perform dynamic light scattering (DLS). The sample in this experiment was the VSD in DMPC solution prepared as described above at a concentration of ~40 μM. Sample was placed into a 1 cm quartz cuvette, and fixed-angle DLS was performed at 25 °C. Measurements of fixed-angle scattering were averaged over time intervals and, therefore, gave the correlation function, which in turn yielded the particle size distribution (assuming exponential correlation), reported as diameter in nm. This processing was done using the NanoBrook instrument analysis software. Particle size analysis assumes spherical geometry.

### 4.6. Recombinant Production of ProTx-II

ProTx-II (UniProt ID P83476) was biosynthesized using a procedure published by Blumenthal et al. [32]. In this procedure, the ProTx-II was expressed with an N-terminal maltose-binding protein (MBP) fusion partner and an enterokinase cleavage site between them. The fusion protein was expressed in BL21(DE3) cells, and cells were lysed by sonication. The fusion protein was purified by Ni^2+^ affinity chromatography. Fractions containing fusion protein as detected by SDS-PAGE analysis were pooled and used for refolding. Before oxidative refolding, the sample was incubated with 10 mM dithiothreitol (DTT) at 4 °C for 16 h. This was followed by dialysis against 2.5 mM reduced glutathione (GSH), 50 mM TRIS pH 8.0 and 150 mM NaCl. The dialyzed solution was then supplemented with oxidized glutathione (GSSG) by dropwise addition of concentrated GSSG solution to a final concentration of 0.5 mM and incubated at 4 °C for 72 h. The resulting solution was dialyzed against 50 mM NaHCO_3_ and cleaved by the addition of 10 U enterokinase (New England Biolabs, Ipswich, MA, USA). After overnight cleavage at 25 °C, the ProTx-II was purified by reverse-phase HPLC using a C-18 Zorbax column (Agilent, Santa Clara, CA, USA). The fraction containing refolded ProTx-II (confirmed by mass spectrometry) was lyophilized, and the solid peptide was stored at −20 °C until needed.

### 4.7. FMOC Peptide Synthesis and Refolding of GpTx-I

GpTx-I toxin (UniProt ID P0DJA9, residues 47–80, without C-terminal amidation) was synthesized using florenyl methyloxy carbonyl chloride (FMOC) protection group-based peptide synthesis. Coupling reactions were performed using an Applied Biosystems ABI 433A peptide synthesizer. The following FMOC amino acids were used: Fmoc—Arg(Pbf)—OH, Fmoc—Asn(Trt)—OH, Fmoc—Asp(OtBu)—OH, Fmoc—Gly—OH, Fmoc—His(Trt)—OH, Fmoc—Ile—OH, Fmoc—Leu—OH, Fmoc— Lys(N^ε^-Boc)—OH, Fmoc—Met—OH, Fmoc—Phe—OH, Fmoc—Pro—OH, Fmoc—Ser(tBu)—OH, Fmoc—Thr(OtBu)—OH, Fmoc—Val—OH, Fmoc—Tyr(OtBu)—OH, Fmoc—Cys(Trt)—OH, Fmoc—Trp(Boc). Amide (MBHA) resin was used to couple the first amino acid of the peptide. Synthesis was performed on a 0.12 mmol scale. Activation of carboxyl groups was performed using 1-hydroxybenzotriazole (HOBt), and n-methyl pyrrolidine (NMP) was used as the reaction solvent. The synthesizer used six solutions in the synthesis program that were prepared just prior to the reaction. Solution 1 was prepared using 19 mL acetic anhydride, 9 mL diisopropylethylamine (DIEA) and 0.8 g HOBt and brought to a total volume of 300 mL using NMP. Solution 2 was prepared by combining 13.51 g HOBt and 37.9 g of hexafluorophosphate benzotriazole tetramethyl uronium (HBTU) and bringing it to 200 mL with dimethylformamide (DMF). Solution 3 was prepared with 69 mL of DIEA brought to 200 mL with NMP. These solutions were used in the activation and coupling steps. Deprotection of FMOC groups was performed using piperidine. Solution 4 was comprised of piperidine only. Solution 5 was pure NMP, and solution 6 was pure methanol (as washing solvents). The program was run, and the synthesis proceeded for roughly 36 h. After side chain protection group cleavage and cleavage from the resin with trifluoroacetic acid (TFA) containing triethyl silane (TES), the crude peptide was precipitated using diethyl ether. The resulting crude peptide was dissolved in water with 0.1% TFA and then purified via reverse-phase HPLC using a 0–100% acetonitrile/water gradient (with 0.1% TFA) over 30 min with a flow rate of 3 mL/min using an Agilent C18 semi-preparative HPLC column with inner diameter of 9.6 mm. About 400 mg of the linear peptide toxin was obtained per HPLC run. Then, 10 mg of the purified toxin was subjected to oxidative refolding in accordance with a published protocol [8]. The HPLC-purified toxin solution was diluted from roughly 15 mL to 4 L with refolding buffer. The refolding buffer contained 3.3 L of HPLC-grade water, 300 mL of acetonitrile, 2.0 g of GSSG, 1.0 g of GSH and 400 mL of TRIS pH 7.5. The solution was incubated overnight at room temperature while stirring. The refolded peptide was then concentrated by ion-exchange chromatography (IEX) using a 5 mL SP (sulfonyl propyl) column (Cytiva, Marlborough, MA, USA). Prior to IEX, the pH of the peptide solution was lowered to 4.0 using glacial acetic acid. The IEX was performed using a linear gradient from 20 mM sodium acetate pH 4.0 as buffer A to 1 M NaCl as buffer B. The IEX run contained only one peak, which corresponded to refolded GpTx-I. The peak fractions were pooled and directly used for injection into HPLC and purified using the same protocol as for the linear peptide above. This yielded ~7mg of refolded toxin, which was then lyophilized and stored at −20 °C until used.

### 4.8. Microscale Thermophoresis

MST experiments were performed using a Monolith NT.115 (NanoTemper, Munich, Germany) instrument. Peptide toxins ProTx-II and GpTx-I were labeled with Alexa Fluor 488-NHS dye (Invitrogen, Waltham, MA, USA). The labeling reactions were performed in 20 mM HEPES pH 8.0 for 30 min. Unreacted free dye was separated from labeled peptides using Sephadex G-25 PD-10 gravity desalting columns (Cytiva, Marlborough, MA, USA). The resulting fractions were analyzed by MALDI mass spectrometry to confirm labeling. Binding titrations were performed by titrating varying amounts of VSD/DMPC into 50 nM of labeled toxin using a 50 µM VSD stock solution. Titrations included 16 VSD concentration steps ranging from ~0.5 nM to ~10 μM. The resulting data were analyzed using either the instrument analysis package or Palmist software [19] to calculate *K*_d_ values for each titration.

## Figures and Tables

**Table 1:** Summary of MST fitting parameters and results.

Sample	*K*_d_ (nM)	Conf. Int. (nM)	Repeats	F_B *_ (‰)	*F*_AB *_ (‰)	rmsd (‰)
Na_V_1.7 VSD2 in DMPC + ProTx-II	200	160–250	2	956.6	981.9	1.283
DMPC control + ProTx-II	50,000 *	10,000–∞	2	974.5	984	1.318
Na_V_1.7 VSD2 in DMPC + GpTx-I	700	300–1500	2	977.8	985.8	1.369
Incompletely refolded Na_V_1.7 VSD II + GpTx-I	n/a	n/a	2	n/a	n/a	n/a
F813A Na_V_1.7 VSD2 in DMPC + GpTx-I	1300	600–3000	1	986.1	988.5	0.225
D816A Na_V_1.7 VSD2 in DMPC + GpTx-I	5000	3000–15,000	1	981.7	993	0.636

All MST data were fit in the Palmist software, and 63.8% confidence intervals calculated based on error-surface projection [19]_._ *F*_B *_ is the normalized relative fluorescence of free toxin, and *F*_AB *_ the normalized relative fluorescence of bound toxin. rmsd is the root mean square deviation of the measured data points from the fitted binding curve. For incompletely refolded VSD2, no binding was observed. * The *K*_d_ for DMPC control is an apparent *K*_d_ value based on the equivalent protein concentration dissolved in the lipids (see Figure 3).

## Data Availability

Raw MST data can be obtained from the corresponding author upon request.

## Supplementary Materials

The following are available online at www.mdpi.com/2072-6651/13/10/722/s1, Figure S1: Size-exclusion chromatography of the TrpΔLE–VSD2 fusion protein after hydroxylamine cleavage. Figure S2: CD spectrum of Na_V_1.7 VSD2 in DMPC solution. Figure S3: MALDI-TOF analysis of GpTx-I labeling with NHS-reactive Alexa Fluor 488 dye. Figure S4: Absorbance spectrum of incompletely refolded Na_V_1.7 VSD2 in DMPC solution. Figure S5: Overview and details of the toxin binding interface in the X-ray crystal structure of Na_V_AB/Na_V_1.7 chimera–ProTx-II complex (PDB ID 6N4I). Figure S6: Overlay of the MST binding curves of WT and two mutants of Na_V_1.7 VSD2 in DMPC vesicles with GpTx-I.
