# Supplementary material for: Expression, Purification and Refolding of a Human NaV1.7 Voltage Sensing Domain with Native-like Toxin Binding Properties
Source: Toxins (Basel). 2021 Oct 12;13(10):722. doi: 10.3390/toxins13100722 (PMC8541342; doi:10.3390/toxins13100722)
Supplement: Supplementary file 1 [file toxins-13-00722-s001.zip › toxins-1399858-supplementary.pdf]

toxins

MDPI

Supplementary Materials:

Expression, Purification and Refolding of a Human NaV1.7 Voltage Sensing Domain with Native-Like Toxin Binding Properties

Ryan V. Schroder, Leah S. Cohen, Ping Wang, Joekeem D. Arizala and Sébastien F. Poget

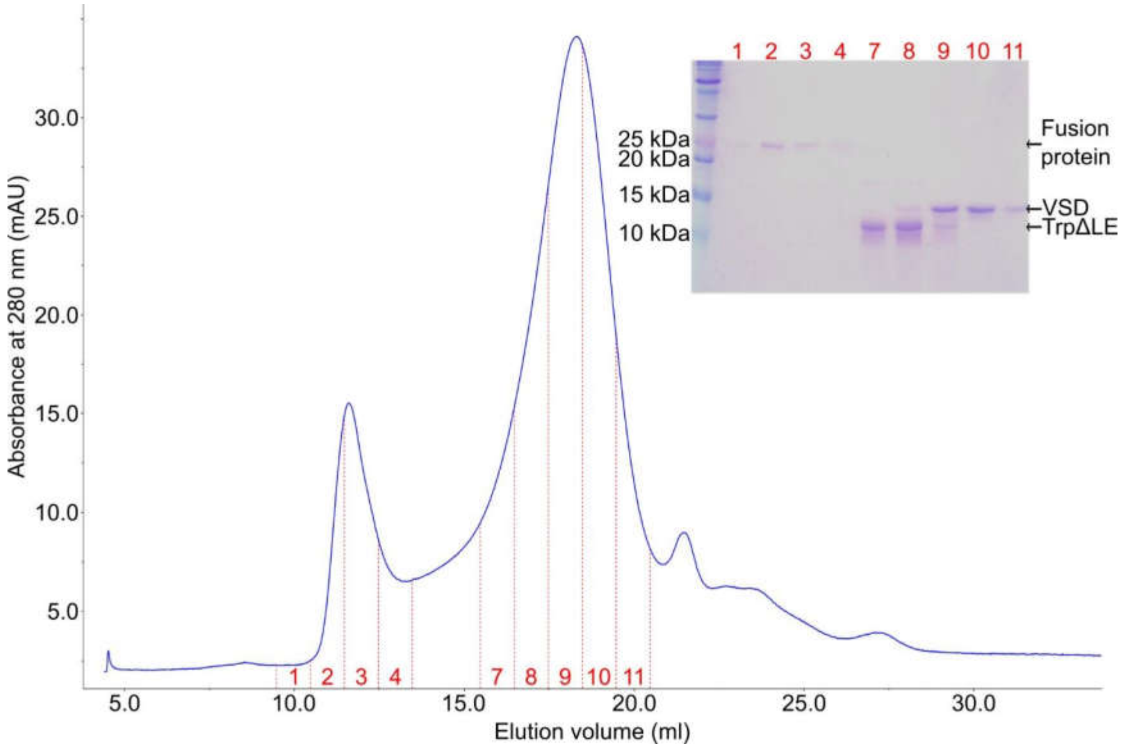

Figure S1. Size-exclusion chromatography of the TrpΔLE-VSD2 fusion protein after hydroxylamine cleavage. The chromatogram shows the result of the purification on a Superdex 200 (Cytiva) size exclusion column using a 1% SDS running buffer. The analyzed fractions are indicated on the chromatogram and labeled with the corresponding numbers on the SDS-PAGE gel shown on the upper right side. Fractions 1–4 contain unreacted fusion protein. Fractions 7–11 contain the VSD (upper band) and TrpΔLE (lower band), which are eluted in an overlapping peak on SEC. Upon reconstitution of the VSD into DMPC vesicles by dialysis, any remaining TrpΔLE contaminant precipitates out, yielding pure VSD.

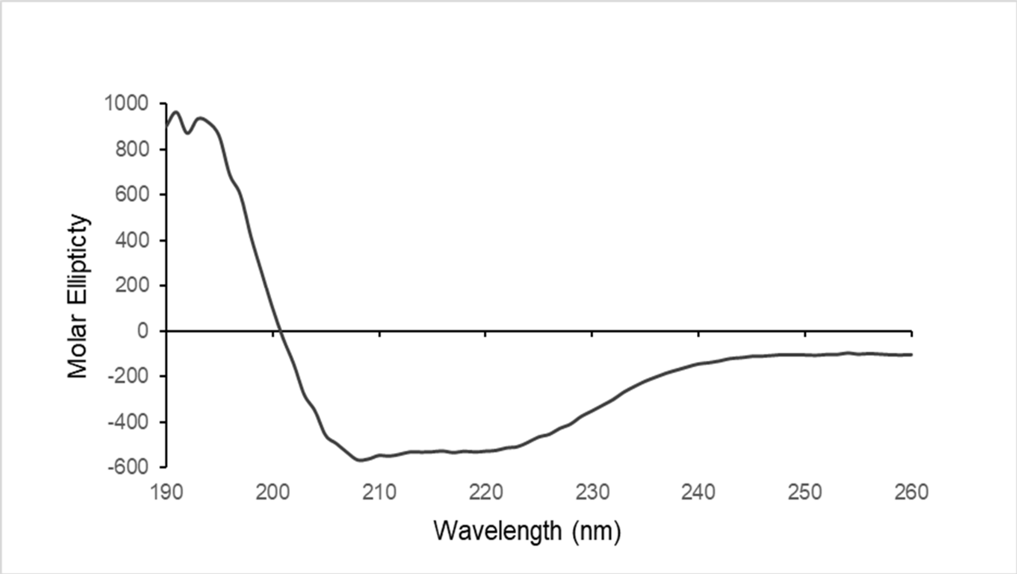

Figure S2. CD spectrum of Nav1.7 VSD2 in DMPC solution. The spectrum displays the local minima at 208 and 222 nm indicative of proteins containing mainly α-helical secondary structure.

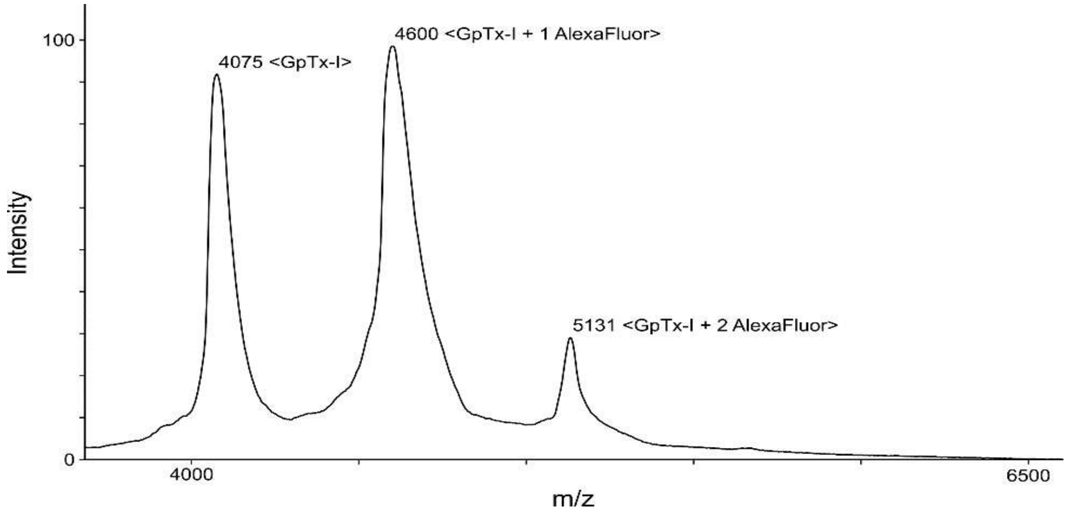

Figure S3. MALDI-TOF analysis of GpTx-I labeling with NHS-reactive Alexa Flour 488 dye. The labeled toxin fraction shows the presence of unlabeled, single-labeled and double-labeled toxin, based on the theoretical toxin mass of 4074.9 Da and the expected mass change of +528 Da for the addition of each fluorescent dye.

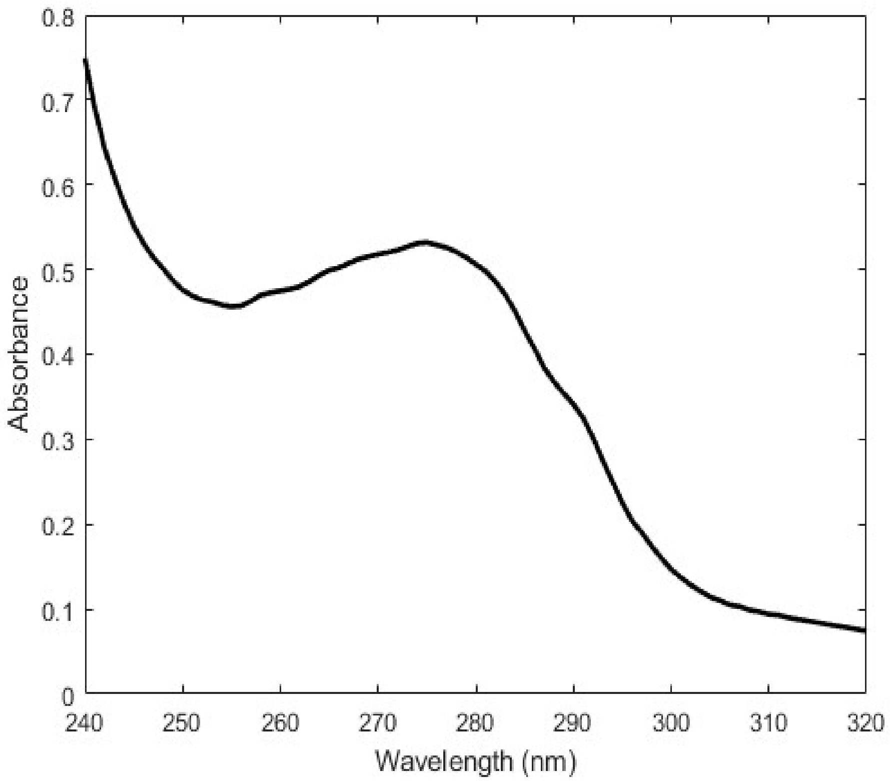

Figure S4. Absorbance spectrum of incompletely refolded Nav1.7 VSD2 in DMPC solution. The absorbance maximum at 280 nm demonstrates that the DMPC aggregates in this partially refolded sample contain protein, and are therefore a good negative control to demonstrate the specificity of the observed toxin binding in the fully refolded VSD samples.

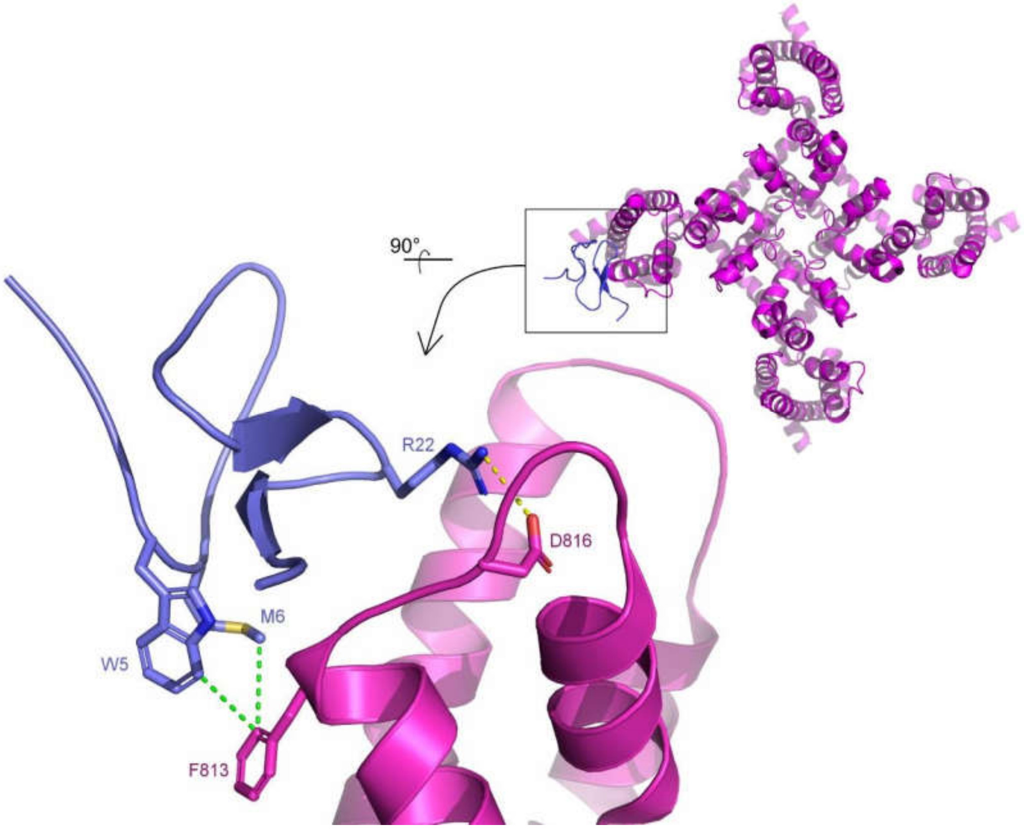

Figure S5. Overview and details of the toxin binding interface in the X-ray crystal structure of NavAB/Nav1.7 chimera-ProTx-II complex (PDB ID 6N4I). The channel is shown in magenta, and the toxin in blue. The overview picture on the top right shows the channel when looking down at the membrane plane from the extracellular side. Only one of four bound toxins is shown for clarity (since this chimeric channel has 4 VSD2-subunits, 4 bound toxins are found in the crystal structure). In the expanded view, the side chains of the two residues F813 and D816 in the VDS2 S3-S4 loop that were mutated in this study are shown in stick representation. The side chains of interacting residues on the toxin are shown as sticks, with specific interactions shown in yellow (electrostatic) or green (hydrophobic) dashed lines. When the sequences of ProTx-II and GpTx-I are compared, a conserved W to F mutation is found in position 5 and M6 is conserved. While the similarity in the second half of the GpTx-I sequence is lower, an arginine is present at the position equivalent to 23 in ProTx-II. This suggests that interactions of similar nature are involved in GpTx-I and ProTx-II binding, a hypothesis that is supported by the results of our mutation studies. Figure generated using Pymol (Schrödinger LLC).

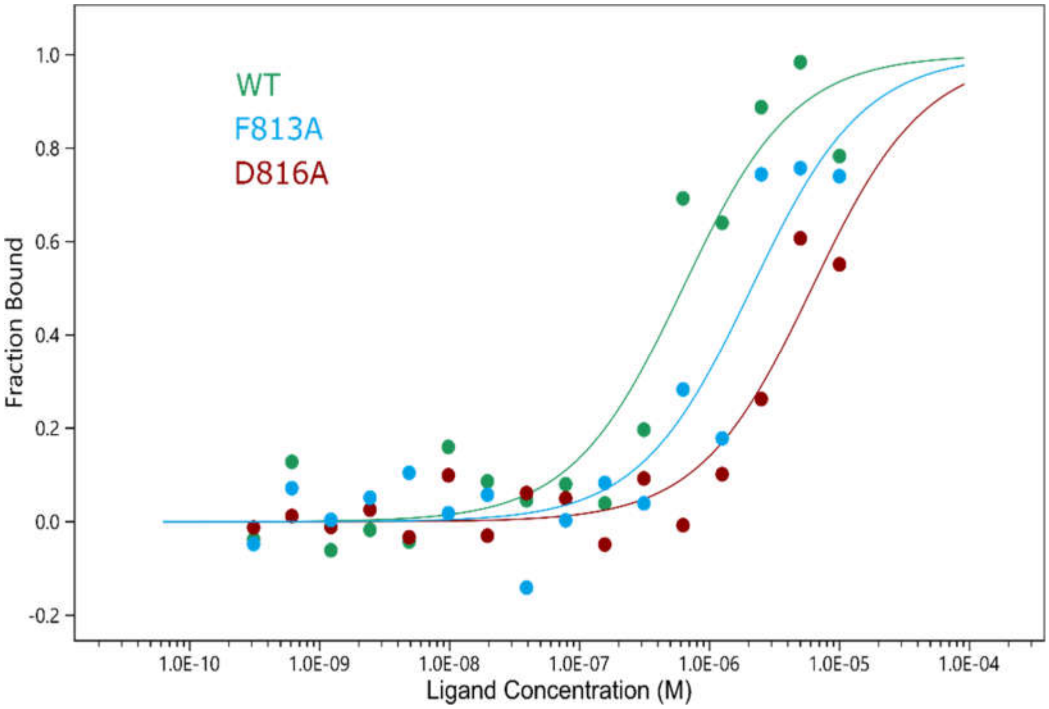

Figure S6. Overlay of the MST binding curves of WT and two mutants of Nav1.7 VSD2 in DMPC vesicles with GpTx-I. The analysis of the MST binding titrations was carried out with the NanoTemper analysis software, and the relative fluorescence measurements and fitted binding curves were combined into a single graph to illustrate the rightshift (lower binding affinity) in both mutants. Based on this analysis, both Phe 813 and Asp 816 appear to play an important role in binding.
